# The Effects of Bilingualism on the Executive Control Abilities of the Prader-Willi Syndrome Population

**DOI:** 10.1007/s10936-024-10123-3

**Published:** 2025-03-12

**Authors:** Estela Garcia-Alcaraz, Juana M. Liceras

**Affiliations:** 1 Department of Spanish, Modern, and Classical Languages, University of the Balearic Islands/Universitat de les Illes Balears, Ramon Llull, Ground floor (office AB09), Cra. de Valldemossa, km 7.5, Palma (Illes Balears), C.P. 07122 Spain; 2 Faculty of Arts, Department of Modern Languages and Literatures & Department of Linguistics, University of Ottawa and Nebrija University, University of Ottawa, Room #217, 70 Laurier East, Ottawa, ON K1N 6N5 Canada

**Keywords:** Bilingualism, Genetic disorders, Prader-Willi syndrome, Executive control

## Abstract

Unlike with the typically developing population, non-typically developing individuals, especially those with intellectual disabilities, have usually been recommended to learn and use only one language, despite perhaps coming from bilingual families or living in multilingual environments. This common practice, however, is not backed by empirical evidence; previous research, although limited, has systematically shown that bilingualism does not have negative effects. This study investigates how bilingualism shapes the executive control abilities of individuals with genetic disorders. Specifically, we compare the interference suppression abilities of Spanish–Catalan bilinguals and Spanish monolinguals with Prader–Willi syndrome. Fifteen participants with Prader–Willi syndrome were recruited in Spain. The bilingual group consisted of seven Spanish–Catalan bilinguals from Catalonia—an officially bilingual territory of Spain—, while the monolingual group was formed by eight Spanish speaking individuals from Madrid—an officially monolingual territory. Participants were administered two widely used psychological tasks: the Flanker Task (a non-language-based task) and the Stroop Task (a language-based task). Three experimental conditions were included in each task: neutral, congruent, and incongruent. Both accuracy and reaction time data were collected and analyzed. The results obtained are consistent between both tasks in showing (i) no detrimental effects of bilingualism; (ii) a high answer accuracy rate; (iii) a practice effect (the more familiar participants became with the tasks the faster their answers became); (iv) sensitivity to an interference effect (higher reaction times for incongruent trials than neutral trials) but not to a clear facilitation effect (lower reaction times for congruent trials than neutral trials). These results, far from being anecdotal, are in line with results from previous research investigating the effects of bilingualism among typically developing individuals as well as non-typically developing individuals with and without genetic disorders. This study not only evidences that Prader–Willi individuals can become bilingual if they are exposed to more than one language, but also that they can do so without showing negative effects at the cognitive level. In fact, taking into account the trend in the descriptive data, if an effect of bilingualism were in place, it would be a positive one. Bilingualism has repetitively been proven to neither be a burden nor to have negative effects for the typically or the non-typically developing population. Thus, as previous researchers have pointed out, there seems to be a clear incongruity between what the research is showing and the actual advice that these individuals and their families are receiving, and this should be amended without further delay.

## Introduction

Today, more than half of the world’s population speaks more than one language (Grosjean, 2022). Therefore, it is not surprising that in the past few decades the study of bilingualism has become a fruitful field of research, due, in part, to researchers’ interests in examining the effects of bilingualism on speakers’ linguistic and cognitive abilities. Previous literature suggests that speaking two languages may come with some costs (e.g., crosslinguistic transfer, a reduced vocabulary in each language when compared to monolinguals, and/or a certain delay in the language acquisition process) but also with some benefits (e.g., enhancement of different cognitive abilities) (Adesope et al., 2010; Ardila, 2012; among others). However, independently of the potential effects of bilingualism, there is a consensus that bilingualism is important beyond the linguistic and cognitive implications that it may entail: speaking more than one language also has positive ramifications at the personal, affective, social, and professional levels (Bialystok, 2016). As a result, bilingualism has been traditionally defended and encouraged among the typically developing population (TD). However, in non-TD (non-TD) individuals, bilingualism has usually been discouraged, or, rather, there has been an absence of encouragement in becoming bilingual (Kay-Raining Bird et al., 2012; Marinova-Todd et al., 2016; among others). Individuals with developmental disabilities (DD)—especially those also entailing intellectual disabilities (ID), as may be the case in individuals with certain genetic disorders—have been frequently dissuaded from becoming bilingual under the argument that adding a second language could potentially affect them negatively by having counter-productive effects on their first language development (Cleave et al., 2014; Peña, 2016). Nevertheless, this practice seems unfounded since previous research has shown that non-TD individuals with and without ID do become bilingual if they are given the opportunity, and they are able to do so without bilingualism having a detrimental effect (see Kay-Raining Bird et al., 2016 for an overview). This paper aims to contribute to this limited but growing body of research by investigating the effects of bilingualism on the executive control abilities of individuals with genetic disorders. More specifically, we analyze the interference suppression abilities of Spanish–Catalan bilinguals and Spanish monolinguals with Prader–Willi syndrome (PWS), a virtually non-explored population so far. This research not only contributes new empirical data to the field, but also provides families and intervention professionals working with this population with valuable scientific results that may help them to make informed decisions about these individuals’ becoming bilingual when being raised in a bilingual/multilingual family and/or environment.

### The Effects of Bilingualism on the Executive Control Abilities of Bilingual Speakers

#### Typically Developing Individuals

Executive control (EC)—also referred to in the literature as executive function or cognitive control (Diamond, 2013, p. 136)—refers to those “general-purpose control mechanisms that enable people to regulate their thoughts and behaviors to align with their goals” (Friedman, 2016, p. 535). More specifically, and following Sekerina et al. (2019, p. 2), these mechanisms “refer to those cognitive processes that integrate, regulate, and control other cognitive processes, processes such as planning, inhibiting, shifting (from one task or rule to another), and updating (stored material with new material)”. Among the different EC abilities, inhibition has received a considerable amount of attention because it is considered a relevant means for testing a potential beneficial effect of bilingualism. Bilinguals, due to their experience handling two languages, are expected to show a certain advantage over monolinguals when asked to override distracting information, as they are trained to deal with two linguistic systems (see Adesope et al., 2010; among others). However, as different researchers point out, the term inhibition has been used indistinctively to refer to two different processes: “response inhibition” and “interference suppression” abilities (see Luk et al., 2010; Traverso et al., 2018). In Brydges et al.’s (2013, p. 1) words, while the former refers to “the suppression of a prepotent or automatic behavioral response” the latter denotes “the ability to control for distracting stimuli or information due to stimulus competition”. It is in this latter one—interference suppression abilities—where a potential bilingual advantage has been said to arise.

Different experimental tasks have been used within the field to analyze the interference suppression abilities of TD bilinguals in comparison to their monolingual peers. In this study we analyze those abilities by administering two of the most popular and extensively used tasks: the Flanker task (Eriksen & Eriksen, 1974) and the Stroop task (Stroop, 1935). The Flanker task is a non-language-based experiment which presents different sequences of arrows/chevrons and where participants are asked to determine the direction of the central arrow/chevron. A variation of this task is the Attentional Network Test (ANT-Fan et al., 2002), in which a Flanker task and a Cue reaction time task are combined (Posner, 1980). On the other hand, the word-colour Stroop task is a language-based experiment that presents different colour words, and participants are asked to determine the ink colour of the words. In both tasks, participants are typically presented with congruent and incongruent trials, although it is also common to include neutral trials. Participants only experience conflict with incongruent trials, for which they need to resort to their interference suppression abilities to resolve them satisfactorily.

Hilchey and Klein (2011) frame the positive effects of bilingualism under two hypotheses: the “*Bilingual Inhibitory Control Advantage*” (BICA hypothesis) and the “*Bilingual Executive Processing Advantage*” (BEPA hypothesis). These two hypotheses were originally proposed in relation to the results of nonverbal EC tasks, but different researchers have extrapolated them to EC language driven tasks (Coderre & Van Heuven, 2014; Vinerte, 2018). The difference between both proposals lies in the fact that while in the BICA hypothesis bilinguals are only expected to outperform monolinguals when resolving conflict (i.e., performing better when resolving incongruent trials), under the BEPA hypothesis bilinguals are expected to excel across the board and not only when solving conflict. Different studies, either explicitly or implicitly, have put these hypotheses to the test.

We will now review very briefly the effects of bilingualism when assessing the EC abilities of monolingual and bilingual speakers using a Flanker task/ANT task, and the Stroop task. Focusing on children first, different studies have reported positive effects of bilingualism when administering the Flanker/ANT tasks (Calvo & Bialystok, 2014; Engel de Abreu et al., 2012; Kapa & Colombo, 2013; Yang et al., 2011; among others) and the Stroop task (principally nonverbal versions/adaptations due to the necessity of reading skills not always mastered by a given age) (Esposito et al., 2013; Poulin-Dubois et al., 2011; among others). However, it is important to highlight that the neutral effects of bilingualism have also been reported at the EC level when administering the Flanker/ANT tasks (Antón et al., 2014; Bialystok et al., 2010; among others) or the Stroop task (Duñabeitia et al., 2014; among others). Similarly, among the adult population we also find mixed results. On the one hand, better outcomes at the EC abilities have been detected when administering the Stroop task (Bialystok et al., 2008, 2014; among others) and the Flanker/ANT tasks when analyzing behavioural data (Luk et al., 2011; Pelham & Abrams, 2014; among others), electrophysiological measures (Kousaie & Phillips, 2012; among others) and event-related functional magnetic resonance imaging technology results (Abutalebi et al., 2012; Luk et al., 2010). Likewise, different studies have reported neutral effects of bilingualism for both the Stroop task (Ryskin et al., 2014) and the Flanker/ANT (Costa et al., 2009). Nevertheless, Costa et al. (2009) defend that in order to see a positive effect of bilingualism among the young adult population—a highly targeted population in previous studies—researchers need to present them with particularly challenging tasks, a position also supported by Grundy et al. (2017). It is beyond the scope of this paper to carry out a detailed analysis of the different factors that could have an impact on the relation between bilingualism and EC (see Valian, 2015 for an overview). Notwithstanding, one factor that has been widely scrutinized is age, since previous literature shows that bilingualism effects are easier to capture at earlier or later ages given that, during young adulthood, individuals would either be at their top processing level (Kroll & Bialystok, 2013) or exposed to different highly demanding cognitive activities providing similar outcomes than bilingualism (Valian, 2015).

Be this as it may, findings supporting the positive effects of bilingualism have led different researchers to advocate for the existence of a bilingual advantage in terms of cognitive performance (Bialystok et al., 2012). The acceptance of the existence of the so-called bilingual advantage has remained practically undisputable for many years. However, during the last decade, different critical voices have emerged either questioning the actual existence of such an advantage (Paap et al., 2015; Paap & Greenberg, 2013) or suggesting that it could be somehow the result of a publication bias (de Bruin et al., 2015). Recently, Leivada et al. (2021) have contributed to this discussion by stating that it could be premature to draw conclusions from the information at hand and that more research is needed to have a clearer picture of the phenomenon. The authors propose avoiding future research from a dichotomous perspective (i.e., based on a presence/absence of a uniform bilingual advantage) and rather focusing instead on the different factors (e.g., task effects, sample size, type of bilingualism, etc.) that may play a role on *bilingual effects*, which is the expression proposed by these authors as a substitute to the controversial *bilingual advantage*. We will thus adhere to the bilingual effects terminology.

#### Non-Typically Developing Individuals

The study of the effects of bilingualism on the non-TD population, in contrast to the TD population, is relatively recent, and, although it is a growing body of research, the available information is still scarce. Previous research within this field has mainly focused either on individuals with Developmental Language Disorder (DLD) or with Autism Spectrum Disorder (ASD). Results from these populations are only partly relevant to our study because individuals with DLD do not have intellectual disabilities (ID) and those with ASD that have IDs are normally excluded from the experimental sample. Thus, the study of the effects of bilingualism among individuals with ID is practically nonexistent and the little we know is limited almost entirely to the Down syndrome (DS) population.

Research aimed at investigating how monolinguals with DLD and ASD compare to TD monolinguals in terms of EC abilities has shown some shortcomings for the DLD and ASD groups (see Marton et al., 2019 for an overview on DLD and Nadig & Gonzalez-Barrero, 2019 on ASD). However, within bilingualism studies, researchers are interested in disentangling whether bilingualism may shape the EC abilities within the idiosyncrasies of non-TD individuals (see for example Peristeri et al., 2020). Similar to the TD population, the limited previous studies at hand suggest either a positive effect (Baldimtsi et al., 2016; Gonzalez-Barrero & Nadig, 2019; Peristeri et al., 2020; among others) or a neutral effect of bilingualism (Laloi et al., 2017; Montgomery et al., 2022; among others). In the specific case of individuals with DS, the results obtained seem to align with those shown by the TD and non-TD populations previously described: (i) positive effects (Pinto-Cardona, 2017) or (ii) neutral effects (Edgin et al., 2011; Martin, 2017). Thus, no negative effects of bilingualism have consistently been found. The question we pose is whether these results will also hold for the PWS population.

### Overview of the Cognitive Abilities of the Prader–Willi Syndrome Population

PWS is a neurodevelopmental genetic disorder not related to geographical origin, socioeconomic status, race, or sex (Alexander et al., 1995) that affects one out of 20,000–25,000 births (Whittington & Holland, 2017). PWS is related to abnormalities in the 15q11.2-q13 region of chromosome 15 due to the absence of paternal expression. As Cassidy et al. (2012) summarize, the lack of the paternal expression of this chromosomal region can be the result of three different mechanisms: *deletion*, which affects 65–75% of individuals and is caused by the absence of the paternal critical region of chromosome 15; *uniparental disomy*, which affects 20–30% of individuals and is the result of not having a paternal copy of chromosome 15 but two maternal copies instead; and *imprinting*, which only affects 1–3% of individuals and is the result of an imprinting defect.

Physically, PWS individuals tend to be of below average stature with facial dysmorphism and decreased muscle tone (hypotonia). However, the most common identifiable trait of the PWS population is their extreme appetite (hyperphagia) (see Cassidy et al., 2012 for a complete review of the clinical characteristics of PWS). Cassidy et al. (2012) identify stubborness, controlling and/or manipulative tendencies, temper tantrums, and obsessive–compulsive behaviours as common behavioural traits. Cognitively, PWS individuals present some shortcomings and delays because they tend to have mild to moderate ID with a full-scale^1^ IQ ranging on average between 50 and 70 (see Dimitropoulos et al., 2013 for a complete overview). In terms of their EC abilities, the scant research at our disposal indicates that, when compared to the TD population, PWS individuals show systematic deficiencies and their EC outcomes seem to be correlated to the invididuals’ IQ (Chevalère et al., 2013, 2015; among others). Regarding bilingualism, no signs of negative effects have been detected for an English–Spanish bilingual with PWS either at the narrative level (García-Alcaraz, 2018) or at the level of grammatical gender representation (Liceras & García-Alcaraz, 2019). More recently, no evidence of a negative effect of bilingualism has been found when comparing the linguistic and metalinguistic abilities of Spanish monolinguals and Spanish–Catalan bilinguals with PWS (Garcia-Alcaraz & Liceras, 2024a). This same lack of a negative effect of bilingualism has been found when comparing, in both languages, the linguistic and metalinguistic abilities of Spanish–Catalan bilinguals with PWS (Garcia-Alcaraz & Liceras, 2024b) and their narrative abilities (Garcia-Alcaraz & Liceras, 2024c).

### The Present Study

The main objective of this study is to analyze the interference suppression abilities of Spanish–Catalan bilinguals in comparison to Spanish monolinguals with PWS (research question 1-RQ1). Additionally, and even though it does not constitute a core aspect of this study, we are also interested in analyzing the global effect of the participants’ individual differences in terms of nonverbal IQ, receptive vocabulary, and sentence recall abilities in the results obtained (research question 2-RQ2).

#### RQ1

Do monolinguals and bilinguals with PWS show different EC abilities, particularly in relation to interference suppression abilities? If they differ, do bilinguals outperform or underperform monolinguals?

#### Hypothesis 1

Based on previous findings concerning the effects of bilingualism on individuals with genetic disorders, a bilingual detrimental effect for the PWS population is not anticipated. If a bilingual effect is detected and it is somehow similar to that described for the TD population (see Sect. “Typically developing individuals”) and, more recently, for non-TD individuals with ASD (see Sect. “Non-typically developing individuals”), this effect is expected to be positive. More precisely, taking into account Hilchey and Klein’s (2011) review of results for the TD population, a potential bilingual effect would manifest itself as a general processing advantage (BEPA hypothesis) rather than as a smaller interference effect (BICA hypothesis).

**RQ2**. To what extent can some of the overall data variance showing the participants’ interference suppression abilities be accounted for by the participants’ individual differences (nonverbal IQ, receptive vocabulary, and sentence recall abilities)?

#### Hypothesis 2

Previous research investigating the EC abilities of the PWS population has indicated that these individuals’ IQ may play a role in their performance (see Sect. “Overview of the cognitive abilities of the Prader–Willi syndrome population”). Therefore, it is possible that despite the lack of overall significant group differences, individual nonverbal IQ differences as well as receptive vocabulary and sentence recall abilities could have an impact on the participants’ EC abilities (RT and accuracy rates).

## Methods

### Questionnaires and Standardized Tests

This study is part of a larger project intended to analyze how bilingualism may shape the cognitive and linguistic abilities of the PWS population. Thus, this project includes additional experimental tasks besides the one presented and detailed in section “Experimental Design”, whose presentation and discussion is beyond the scope of this paper. However, what is of relevance for this study are the standardized tests that were administered to participants to have information on their nonverbal IQ, receptive vocabulary, and sentence recall abilities as well as the sociolinguistic background questionnaires that the participants’ family members were asked to complete to have a better understanding of the participants’ family profile and of the participants’ language use.

Participants’ nonverbal IQ abilities were assessed by administering the *Test of Nonverbal Intelligence 2* (TONI-2) (Brown et al., 1990). This test, which does not require language interaction, includes different abstract problems of increasing difficulty to capture participants’ nonverbal cognitive development. Receptive vocabulary abilities were evaluated using the standardized Spanish version of the *Peabody Picture Vocabulary Test* (PPVT-III) (Dunn et al., 2006). In this test participants were orally presented with words of increasing difficulty and were asked to select the image that best depicted the word heard between four images. Previous research has shown a high correlation between the PPTV and the verbal IQ for TD children and adults (Bell et al., 2001; Hodapp & Gerken, 1999) and individuals with ASD (Krasileva et al., 2017). Thus, the PPVT constitutes a rich measure that goes beyond its primary function. Participants’ sentence recall abilities were measured with the standardized subtest of the Spanish version of the *Clinical Evaluation of Language Fundamentals-CELF5* (Wiig et al., 2018). In this test participants were asked to repeat verbatim different orally presented sentences of increasing difficulty in both length and language. As in the case of the PPVT, it has been suggested that this subtest also goes beyond its primary function of evaluating individuals’ expressive language and is a good indicator for participants’ working memory abilities (Alloway & Ledwon, 2014).

To complement the participants’ cognitive and linguistic information obtained through the three aforementioned standardized tests, their father/mother/legal guardian were asked to complete two questionnaires: a *Family background questionnaire*, and a *Language use and fluency questionnaire*. The former was aimed at giving us an overall overview of the participants (i.e., age, gender, PWS subtype) and a better understanding of the family profile (socioeconomic status measured through the maternal level of education); the latter was aimed at having a better understanding of the participants’ use of language.

### Participants

A total of 15 participants completed the EC experiment: eight Spanish monolinguals (*M* = 22.80 years old, *SD* = 11.58, age range 9;4–47;0) and seven Catalan–Spanish bilinguals (*M* = 18.04 years old, *SD =* 8.95, age range 10;5–33;10). All participants were diagnosed with PWS and no other medical condition potentially affecting their cognitive or linguistic abilities was disclosed. The sample included six females and nine males (11 participants with the Deletion PWS subtype, two with the Disomy subtype and two with the Imprinting subtype). Spanish monolingual speakers were recruited in the Autonomous community of Madrid, a Spanish monolingual region, and bilingual speakers were recruited in Catalonia, a Spanish–Catalan bilingual region. All participants belonged to middle class families.

In the case of the bilingual speakers, given that participants came from principally Catalan speaking families and Catalan was also their language of instruction at school, Catalan was their dominant language, with one exception. These bilingual speakers mostly started their formal contact with Spanish in kindergarten or primary school; therefore, they are early-sequential bilinguals. Participants’ results for nonverbal IQ, receptive vocabulary, and sentence recall abilities are presented in Table 1.

**Table 1:** Nonverbal IQ, receptive vocabulary, and sentence recall measures by group

	Nonverbal IQ		Receptive vocabulary		Sentence recall	
	Monolinguals	Bilinguals	Monolinguals	Bilinguals	Monolinguals	Bilinguals
*Mean*	75.75	90.14	64.13	67.86	66.88	72.14
*SD*	10.35	11.01	16.95	14.50	14.87	20.59
*Minimum*	55	79	55	55	55	55
*Maximum*	87	112	94	91	100	105

Measures are presented in standard scores (*M* = 100, *SD* 15)

In order to analyze both groups’ comparability for the three standardized measures, a mixed-design ANOVA was run with Standardized test as the within-subjects variable (Nonverbal IQ, Receptive Vocabulary, and Sentence Recall) and Group (Monolingual vs. Bilingual) as the between-subjects factor. Results revealed a main effect of Standardized test (*F*(2, 26) = 5.32, *p* = .012, *η*_*p*_^*2*^ = 0.29) but not of Group (*F*(1, 13) = 2.96, *p* = .109, *η*_*p*_^*2*^ = 0.19). The interaction between both factors was not significant (*F*(2, 26) = 0.551, *p* = .583, *η*_*p*_^*2*^ = 0.04). Post hoc pairwise comparisons with the Bonferroni correction showed a significant mean difference between nonverbal IQ and receptive vocabulary abilities (*p* = .015, *d* = 0.86). Thus, these results suggest, on the one hand, that both monolinguals and bilinguals perform comparably in terms of nonverbal IQ, receptive vocabulary, and sentence recall abilities, and, on the other hand, that, in general, PWS individuals show higher nonverbal IQ than receptive vocabulary abilities (a measure highly correlated with verbal IQ). These results are in line with previous research describing the IQ abilities of the PWS population (see Dimitropoulos et al., 2013).

While data from the 15 participants were kept for the Flanker task, data from one bilingual participant were discarded from the Stroop task analysis due to colour blindness. The exclusion of this participant did not affect the comparability of the groups, as the mixed-design ANOVA previously described was rerun without this participant and the results obtained replicated the ones previously described for the complete sample.

### Experimental Design

To measure the participants’ interference suppression abilities, they were administered the Flanker task (nonverbal-based experiment) and the Stroop task (verbal-based experiment). Both tasks were programmed in a single experiment using E-prime software version 2.0 (Schneider et al., 2002a, b). The experimental task was administered in a PC laptop computer (15-inc screen; Windows 7) and participants provided their responses using a portable numpad connected to the laptop via USB. Both RT and accuracy data were collected.

#### Flanker Task

In this task, different chevron sequences were presented, and participants were instructed to determine the direction of the central chevron (right–left). The task included three experimental conditions: (1) **Congruent** (all chevrons pointing to the same direction; < < < < < or > > > > >); (2) **Incongruent** (central chevron pointing to the opposite direction of the other four chevrons; < < > < < or > > < > >); and (3) **Neutral** (a central chevron between two diamonds on each side; ♢♢>♢♢ or ♢♢<♢♢). The task included a total of 96 trials: 32 stimuli per condition (in half of the stimuli the central chevron pointed left and in the other half the central chevron pointed right).

#### Stroop Task

In this task, different coloured words were presented, and participants were asked to determine the ink font colour. The task included three experimental conditions: (1) **Congruent** (matching between the colour word and the colour font. E.g., *Rojo* ‘red’ written with red ink); (2) **Incongruent** (mismatch between the colour word and the colour font. E.g., *Rojo* ‘red’ written with blue ink); and (3) **Neutral** (coloured non-colour words. E.g., *Ventana* ‘window’ written with blue ink). The three stimuli colour words were the Spanish–Catalan translation equivalent for *red*,* blue*, and *yellow* and the three non-colour words were the Spanish–Catalan translation equivalent of the high frequency words of *table*,* leaf*, and *window*. None of the six words included in the experiment are cognates in Spanish and Catalan. The task included a total of 108 trials (36 trials per condition using the red, blue, or yellow ink 12 times within each one). It is important to highlight that given that previous research has shown that multilingual speakers tend to be slower and less accurate when completing the Stroop task in a language other than their L1 (Marian et al., 2013), Spanish–Catalan bilinguals completed the Stroop task in their dominant language. The Catalan and the Spanish tasks were identical except for the language used.

In both tasks all trials were evenly divided in two blocks: two blocks of 48 trials for the Flanker task and two blocks of 54 trials for the Stroop task. To neutralize a potential presentation bias, two lists were created: list A containing block 1 and block 2 (stimuli were presented with order 1–48 for the Flanker task and 1–54 for the Stroop task in each block; and list B containing block 3 and block 4 (exactly the same stimuli of block 1 and 2, respectively, but in the reverse order; i.e., 48/54–1 in both cases). Both lists were counterbalanced across participants.

Trials were presented in sequential order within each block because they were previously pseudo-randomized, ensuring that: (i) no two exact stimuli were presented consecutively; (ii) no more than two trials of the same experimental condition were presented sequentially, and (iii) no more than two left or right answers were presented in sequence (Flanker task)/no more than two of the same ink colour were presented consecutively (Stroop task).

### Procedure

Considering the idiosyncrasies of the PWS population, and tyring to avoid a tiredness and/or boredom effect on the results obtained—especially in the RT data—we decided to administer both tasks in a single EC experiment in which blocks of both tasks were alternating: first a Flanker task block followed by a Stroop task block and then another Flanker task block followed by a Stroop task block. Participants were allowed to take short breaks between blocks.

Before starting with the actual experiment, participants were presented with the overall instructions. Next, they were presented with the Flanker task instructions, and they were asked to complete 12 practice items with corrective feedback. Once participants were ready to start, they completed the first block of the Flanker task without corrective feedback. Then, participants were presented with the Stroop task instructions and were also asked to complete 12 practice trials with corrective feedback. Again, once they were ready to start, they completed the first block of the Stroop task without corrective feedback. When participants had completed a block of each task, they repeated the drill, this second time with a shorter practice session for each task (six practice trials) as they were already familiar with the tasks. While the function of practice trials in the first block was to make sure that participants understood the procedure, their function in the second block was more of a reminder of the task dynamics in order to avoid lower RTs in the first trials of the second block due to a lack of practice. Any doubts that participants may have had were resolved by the researcher when needed.

An example of an incongruent trial is presented for both tasks:

**Fig. 1:**
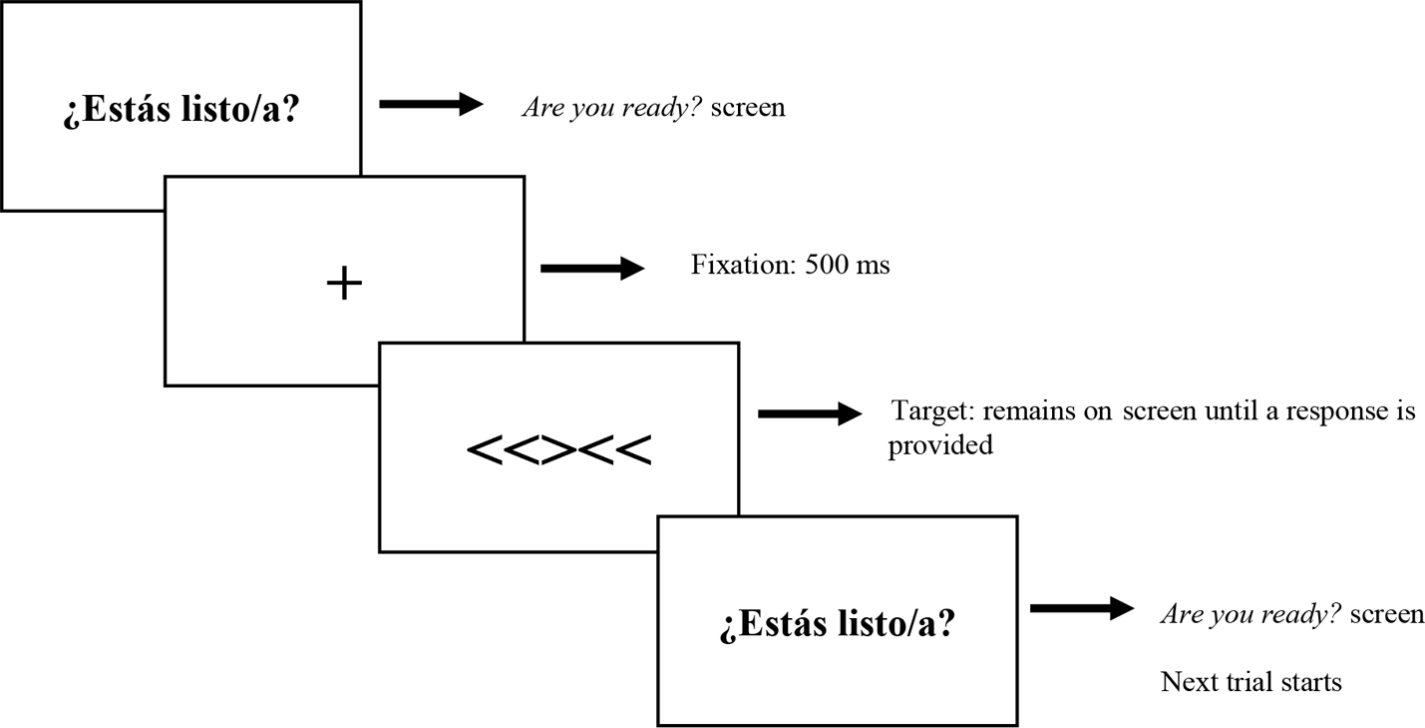
Trial structure for the Flanker task

**Fig. 2:**
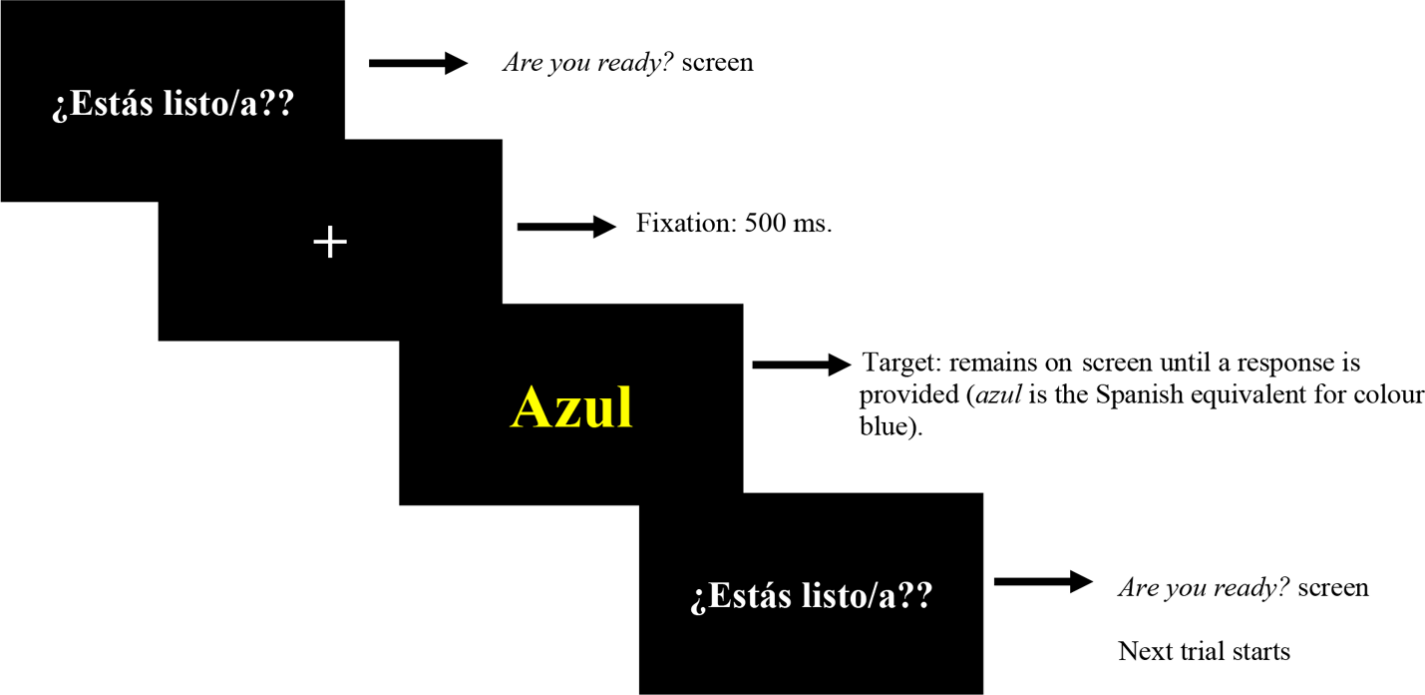
Trial structure for the Stroop task

As shown in Figs. 1 and 2, each trial started with an *Are you ready?* screen. When participants felt ready, they pressed the numpad’s space bar and a central fixation cross appeared on screen for 500 ms followed by the target, which remained on screen until the participant responded. In the case of the Flanker task, target stimuli were presented centered as images with a size of 70% by 35% and participants were instructed to respond as quickly as possible by pressing either button 4 (chevron pointing left) or button 6 (chevron pointing right) on the numpad. In the case of the Stroop task, target stimuli were presented centered on a black screen to ensure good colour visualization in size 60 Times New Roman font. Participants were instructed to respond as fast as possible by pressing either button 4 (red), button 5 (yellow), or button 6 (blue) on the numpad. In both tasks, answer buttons were labelled with stickers to help participants remember the expected button answer for each case. Once participants provided an answer for the target, the sequence started over.

### Data Analysis

Both RT and accuracy data were recorded. However, given that both groups performed practically at ceiling in both tasks for all three experimental conditions (see Table 2), the inferential statistical analysis is focused on the RT data.

Following standard practice within the field, raw data were submitted to a cleaning process as follows: (i) RTs for inaccurate responses were excluded; (ii) RTs below 250ms or above 5000ms were removed; (iii) RTs that were 2.5 standard deviations above or below the mean per condition per participant were discarded. The application of these steps resulted in the removal of 9.65% of the data from the Flanker task (5.76% from the monolingual group and 3.89% from the bilingual group) and 5.55% of the data from the Stroop task (3.30% from the monolingual group and 2.25% from the bilingual group). An inverse transformation (-1000/RT) was applied to the cleaned RT to reduce the typical RT positive skew (Brysbaert & Stevens, 2018).

A mixed-effects model analysis was run in R (version 3.6.2; R Core Team, 2019) using the *lme4* package (version 1.1–23; Bates et al., 2015). The modelling process started with the so-called maximal model (Barr et al., 2013), and, following standard procedures within the field, the model was simplified until convergence was attained. The model simplification process started with the random effects structure by first removing random slope interactions followed by the decorrelation of intercepts and slopes, by the simplification of the item slope structure, and by the simplification of the participant slope structure. The minimal random structure accepted for conducting the analysis was the by-participant and by-item random-intercepts-only. When the random-intercepts-only model did not converge or yielded singularity issues, the fixed effect structure was simplified in a meaningful way to the research objectives set. When convergeance was attained, and while maintaining the random structure constant, the modelling process continued with a simplification of the fixed effect structure (first removing interactions followed by the elimination of individual fixed effects) to check whether exclusion improved the model fit. During the modelling process, all possible options were checked, one at a time, and the new resulting model was compared to the previous one. When the resulting model improved, this was taken as the new comparison baseline until the best-fitting model was achieved. Model comparison was carried out using the second order Akaike Information Criterion (AICc), which is the Akaike Information Criterion (AIC) correction for small samples (Burnham & Anderson, 2002).

The fixed effects included in the maximal model were Block (Block 1, Block 2, Block 3 and Block 4) and the interaction between Experimental Condition (Congruent, Neutral, and Incongruent) and Group (-0.5 for Monolinguals and 0.5 for Bilinguals). The maximal random effect’s structure included intercepts for participants and for items and a slope for the Experimental Condition by participants and slopes for Group and Block by items. Post hoc analysis with Bonferroni correction were run using the *emmeans* package (version 1.4.6) to compare the different levels of the Block variable. The *nloptwrap2* optimizer (Bolker, 2014) was used to enable the models’ convergence (*nloptr* -version 1.2.2.1- package; Johnson, n.d.).

This analysis constitutes phase 1, whose objective was to answer research question one—disentangling whether monolinguals and bilinguals solved interference suppression abilities differently. Additionally, the best-fitting model of phase 1 was submitted to a phase 2 analysis in which the three standardized measures (nonverbal IQ, Receptive vocabulary, and Sentence recall abilities) were z-scored and included as fixed effects. The resulting best-fitting model was then compared to the best-fitting model of phase 1. The objective of this analysis was to answer research question two—whether participants’ individual differences in terms of the three standardized measures played a role in the overall data variance observed. The variance explained by the model was estimated using the *r.squaredGLMM* function (MuMIn package –version 1.43.17; Barton, 2020).

## Results

In this section, descriptive statistics for accuracy data are presented first, followed by descriptive and inferential statistics for the RT data.

**Table 2:** Mean accuracy rate (on a scale of 0–1) by experimental condition, group, and task. SD are reported in parentheses

	Congruent		Neutral		Incongruent	
	Monolinguals	Bilinguals	Monolinguals	Bilinguals	Monolinguals	Bilinguals
*Flanker Task*	0.97 (0.17)	0.98 (0.15)	0.97 (0.16)	0.96 (0.19)	0.90 (0.30)	0.95 (0.23)
*Stroop Task*	0.99 (0.12)	0.99 (0.12)	0.99 (0.08)	1 (0.00)	0.98 (0.13)	0.98 (0.15)

**Fig. 3:**
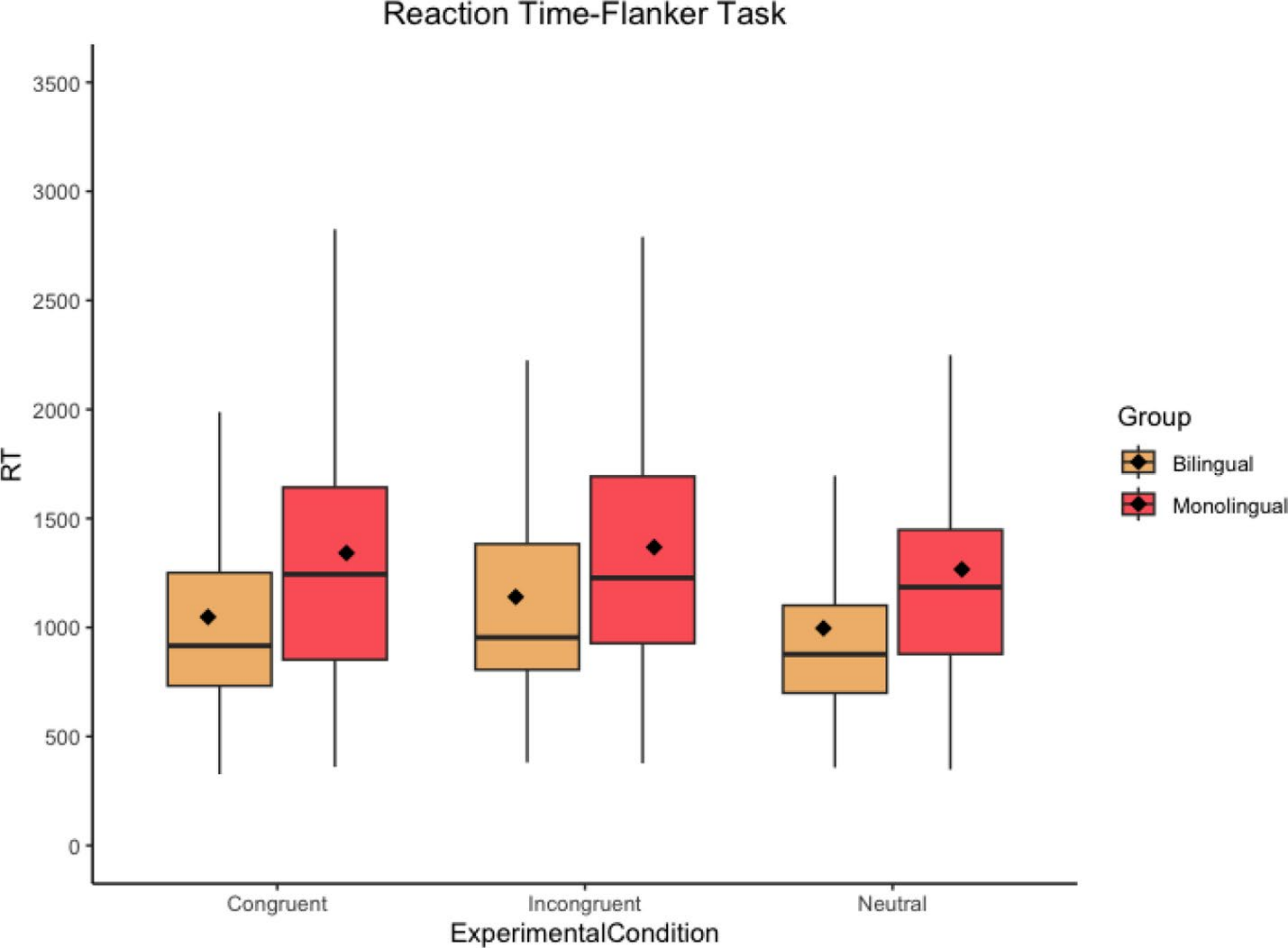
Boxplot for the Flanker task results showing RT (in ms) per group and experimental condition. The black horizontal line indicates the median and the diamond indicates the mean. Outliers are excluded for visibility purposes

**Fig. 4:**
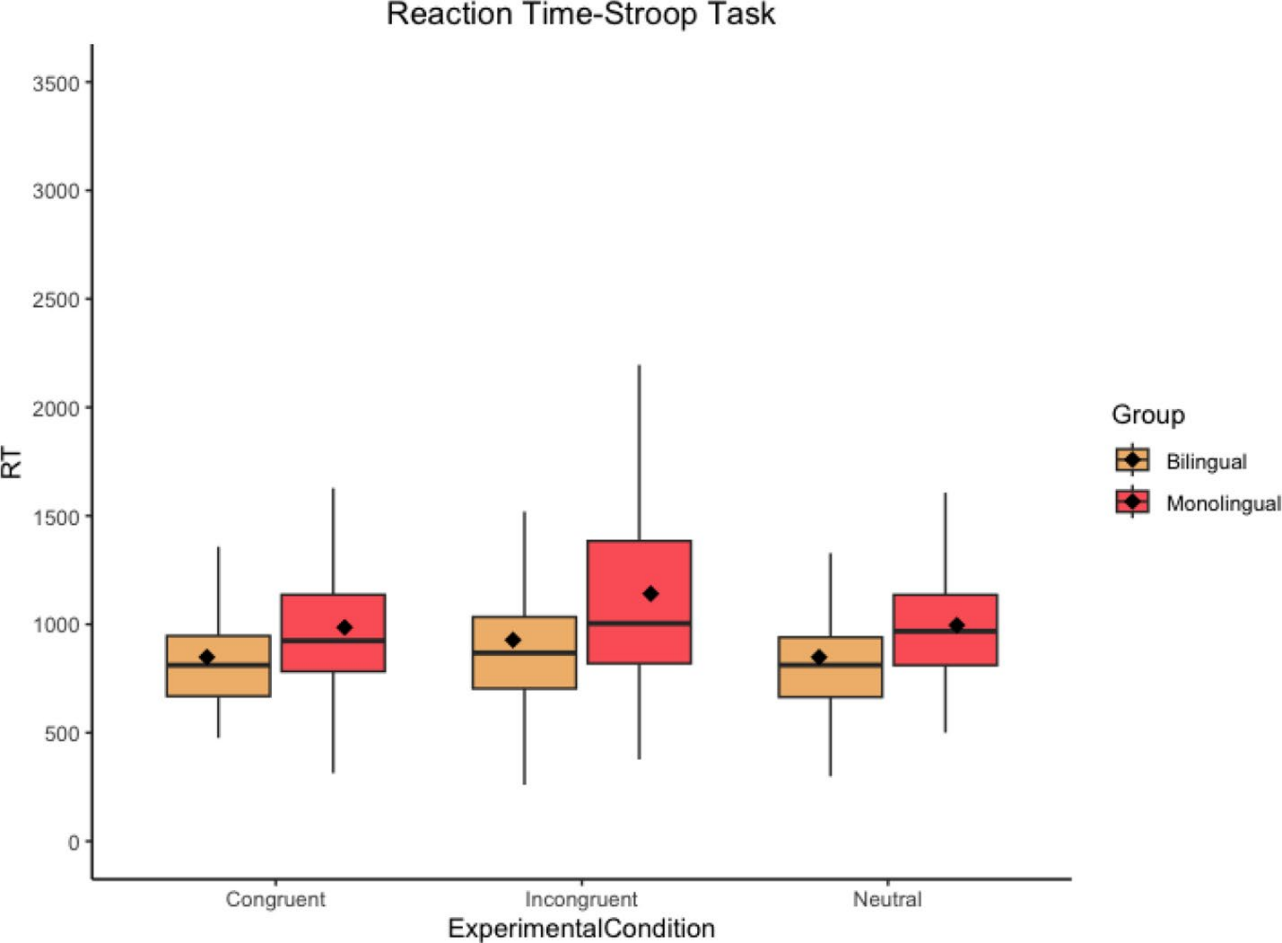
Boxplot for the Stroop task results showing RT (in ms) per group and experimental condition. The black horizontal line indicates the median and the diamond indicates the mean. Outliers are excluded for visibility purposes

Accuracy data show that both groups perform almost at ceiling for all three experimental conditions in both tasks since, on average, participants provided the expected right answer 90–100% of the time. Thus, the understandability of the task was not compromised. Non-transformed RT descriptive data (see Table 3 as well as Figs. 3 and 4) show that, overall, bilinguals are faster than monolingual speakers in all three experimental conditions in both tasks. Likewise, participants, regardless of the group, seem to be sensitive to an interference effect (higher RTs for incongruent trials in comparison to neutral trials) in both tasks but not to a steady facilitation effect (lower RTs for congruent trials in comparison to neutral trials).

**Table 3:** Mean RT (in ms) by experimental condition, group, and task. SD are reported in parentheses

	Congruent		Neutral		Incongruent	
	Monolinguals	Bilinguals	Monolinguals	Bilinguals	Monolinguals	Bilinguals
*Flanker Task*	1427.36 (813.71)	1120.90 (697.08)	1266.15 (594.15)	996.39 (471.15)	1454.47 (760.52)	1153.54 (582.63)
*Stroop Task*	985.76 (333.14)	848.59 (277.56)	995.97 (312.63)	848.46 (285.39)	1140.96 (462.28)	927.66 (349.97)

In order to confirm the inferences drawn, we performed a linear mixed model analysis on the Inverse RT variable for both tasks. We will first focus on the results of the best-fitting model for the Flanker task (Table 4) followed by the results of the best-fitting model for the Stroop task (Table 5).

The Flanker task’s best-fitting model included the fixed effects of Block and Experimental Condition and the by-participant and by-item intercepts. However, the fixed effect of Group did not improve the model fit. For the Experimental Condition predictor, results showed a significant interference effect (*t* = 3.97, *p* = .028) but not a facilitation effect (*t* = 1.45, *p* = .246). In terms of the effect of Block, differences were attested between Block 1 and Block 2 (*t* = -3.81, *p* < .001) and between Block 3 and Block 4 (*t* = 8.91, *p* < .001) but not between Block 1 and Block 3 (*t* = -1.72, *p* = .109) and between Block 2 and Block 4 (*t* = 2.44, *p* = .177).

**Table 4:** Model parameters for the best-fitting model (Flanker task). Dependent variable: inverse RT

Parameters							Random effects			
	Fixed effects						Participant		Item	
	Estimate	SE	t^a^	Pr(>|t|)	Lower limit	Upper limit	Variance	SD	Variance	SD
Intercept	-0.83	0.13	-6.55	< 0.001*	-1.07	-0.58	0.11	0.32	0.001	0.03
Block 2	-0.10	0.03	-3.81	< 0.001*	-0.16	-0.05	-	-	-	-
Block 3	-0.29	0.17	-1.72	0.109	-0.62	0.04	-	-	-	-
Block 4	-0.52	0.17	-3.04	0.009*	-0.85	-0.19	-	-	-	-
Exp. Condition Congruent	0.05	0.03	1.45	0.246	-0.01	0.11	-	-	-	-
Exp. Condition Incongruent	0.14	0.03	3.97	0.028*	0.07	0.20	-	-	-	-
*Post hoc pairwise comparisons* ^*a*^										
Block 2-Block 4	0.42	0.17	2.44	0.177	0.08	0.74				
Block 3-Block 4	0.23	0.03	8.91	< 0.001*	0.17	0.27				

^a^ For Post hoc pairwise comparisons *t* is *t-ratio*

Note. Model reference values for Block: block 1; for Experimental Condition: neutral

The Stroop task’s best-fitting model included the fixed effects of Block, Experimental Condition, and Group as main effects and the by-participant and by-item intercepts. In this case the effect of Group improved the model fit but no main effect was revealed (*t* = -1.54, *p* = .151). Similarly to the Flanker task, results for the Experimental Condition predictor showed an interference effect (*t* = 3.44, *p* = .002) but an absence of a facilitator one (*t* = -0.53, *p* = .601). The effect of Block, as in the case of the Flanker task, yielded significant differences between Block 1 and Block 2 (*t* = -5.08, *p* < .001) and between Block 3 and Block 4 (*t* = 4.59, *p* < .001) but not for Block 1 and Block 3 (*t* = -2.08, *p* = .061) and for Block 2 and Block 4 (*t* = 2.00, *p* = .423). Thus, comparable results were found in both experimental tasks.

**Table 5:** Model parameters for the best-fitting model (Stroop task). Dependent variable: inverse RT

Parameters							Random effects			
	Fixed effects						Participant		Item	
	Estimate	SE	t	Pr(>|t|)	Lower limit	Upper limit	Variance	SD	Variance	SD
Intercept	-1.01	0.09	-11.36	< 0.001*	-1.17	-0.84	0.05	0.23	0.001	0.03
Block 2	-0.11	0.02	-5.08	< 0.001*	-0.15	-0.07	-	-	-	-
Block 3	-0.26	0.12	-2.08	0.061	-0.49	-0.03	-	-	-	-
Block 4	-0.36	0.12	-2.90	0.014*	-0.59	-0.13	-	-	-	-
Exp. Condition Congruent	-0.01	0.02	-0.53	0.601	-0.06	0.04	-	-	-	-
Exp. Condition Incongruent	0.08	0.02	3.44	0.002*	0.03	0.12	-	-	-	-
Group	-0.19	0.12	-1.54	0.151	-0.42	0.04	-	-	-	-
*Post hoc pairwise comparisons* ^*a*^										
Block 2-Block 4	0.25	0.12	2.00	0.423	0.02	0.48				
Block 3-Block 4	0.10	0.02	4.59	< 0.001*	0.06	0.14				

^a^ For Post hoc pairwise comparisons *t* is *t-ratio*

Note. Model reference values for Block: block 1; for Experimental Condition: neutral. Group was contrast-coded (-0.5 for monolinguals and 0.5 for bilinguals)

The marginal R^2^ of the best-fitting model for the Flanker task is 16% and for the Stroop task is 18% and the conditional R^2^ is 57% for the Flanker task and 49% for the Stroop task. This means that while the fixed effects included in the models explained the 16% and 18% of the data variance, respectively, when including the by-participant and the by-item random effects, the explanatory power of the models increased to almost 60% and 50% respectively.

Refitting the best-fitting model of phase 1 to include the three standardized measures did not improve the phase 1 model fit.

## Discussion

This study investigated how the interference suppression abilities of Spanish–Catalan bilinguals and Spanish monolinguals with PWS compared when completing a non-language-based experiment (the Flanker Task) and a language-based experiment (the Stroop task).

Focusing on accuracy data, results revealed a high accuracy rate for both groups. Indeed, the fact that both bilinguals and monolinguals performed almost at ceiling showing a mean accuracy rate for all experimental conditions of ≥ 90% illustrates, on the one hand, that participants understood the task and that they were not providing random answers but thoughtful ones, and, on the other hand, that they did not show signs of their interference suppression abilities being impaired, or at least not impaired with this type of experimental design. High accuracy rates for both the Flanker task and the Stroop task have been also found to be common among the TD young adult population (Grundy et al., 2017; Vinerte, 2018; respectively).

In terms of RT data, results did not reveal a detrimental effect of bilingualism, which is in line with previous research focusing on analyzing the effects of bilingualism at the cognitive level for the DS population (Edgin et al., 2011). However, as in Martin’s (2017) case study for an English–French bilingual with DS, our results did not show a positive effect of bilingualism at the EC domain. These results, far from being anecdotal, are frequent among both the TD population and the non-TD population given that, as discussed in Sect. “Typically developing individuals”, the results reported in the literature are mixed, and the existence of an actual positive effect of bilingualism at the EC domain is a source of debate (see Bialystok, 2016). However, as different researchers defend, not detecting a potential beneficial effect of bilingualism does not necessarily imply its non-existence but rather its difficulty of being isolated under certain circumstances (see Valian, 2015). In fact, if we go back to the descriptive data in Table 3, it suggests a potential positive effect of bilingualism since bilinguals, on average, are faster than monolingual speakers in both experimental tasks for all three experimental conditions; consistent for both the BEPA (faster RT for bilinguals across the board) and the BICA (faster RT for bilinguals when resolving conflict) hypotheses. It is premature to draw conclusions at this stage because inferential statistics did not reveal differences between groups when resolving interference, but, and with all due caution, if we take descriptive data as a mark of likely trend, it could be possible that the suggested differences between groups could be translated into inferential statistics with a larger sample, as small samples could be hindering possible evident differences within and between groups (Leppink et al., 2016, p. 122).

Apart from the similarities of the effects of group between both experimental tasks, similar results are found in both the Flanker and the Stroop tasks for the effects of experimental condition and block. Regarding the first, participants—independently of being bilingual or monolingual—were sensitive to an interference effect but not to a facilitation effect, meaning that, in general, on the one hand, it takes more time for them to resolve conflict than to provide an answer for a neutral item and, on the other hand, they do not significantly “benefit” from the congruency effect of responding to congruent trials faster than to the neutral ones. Again, this pattern is not at all unique and has been previously attested in the TD population because the interference effect is more prominent and stable than the facilitation effect (Kalanthroff & Henik, 2013, p. 413). These results support the accuracy results in terms of showing that participants were doing the task with all their attention because otherwise an interference effect would have probably gone undetected. In terms of the effects of the variable block, once more results mirrored in both experimental tasks displaying a natural and predictable practice effect since the more familiar participants became with the task, the faster they responded to the experimental trials presented. This is evident in that their responses were faster while completing the second block of each task. Analyzing the effects of practice when resolving interference was beyond the scope of this study but it would be advisable for future research to investigate whether more practice for the PWS population could potentially reduce the interference effect in a way like the one that has been suggested for the TD population (see Davidson et al., 2003). Lastly, it is important to highlight that the effect of block is also an indirect measure for the effect of list, as results did not reveal significant differences between blocks 1 and 3 and between blocks 2 and 4 (these pairs of blocks were exactly the same but presented in the reverse order).

Despite previous research having shown EC limitations for the PWS population when compared to the TD population (Chevalère et al., 2015; Woodcock et al., 2009; among others), the effects of bilingualism detected among the PWS population do not seem to differ much from those of the TD population. The comparison between how interference suppression abilities may vary between the PWS population and the TD population was beyond the scope of this study; however, in future research, whether and how they differ should be analyzed. It could be the case that they show similar offline outcomes but different processing characteristics. Also, it would be advisable to include participants with different abilities in terms of nonverbal IQ, receptive vocabulary, and sentence recall abilities to analyze in detail how these abilities may have an impact on the obtained results. We controlled for and included these measures in phase 2 of the analysis without finding a significant effect of any of them. However, it is important to note that despite the individual differences that participants may have, as reported in Sect. “Participants”, both groups were not statistically significantly different in any of the three measures. Thus, it could be possible that, in this respect, more variability among the participants may lead to more information about the potential effects of these abilities. We are aware that the participant sample included in this study is limited but it is important to acknowledge the difficulty of recruiting participants with PWS, particularly bilingual speakers. We need to be cautious with the obtained results and take them as a first approach to the study of the effects of bilingualism among this population, from which we can continue building knowledge.

## Conclusions

This study did not show a detrimental effect of bilingualism among the PWS participants, which is in line with the results of previous research studying the effects of bilingualism in non-typically developing populations with and without genetic disorders (see Kay-Raining Bird et al., 2016). Thus, this study contributes new empirical evidence to debunk the myth of bilingualism being a burden for the non-TD population. As a consequence, it seems paradoxical to encourage and promote bilingualism among the TD population but to do the opposite with the non-TD population without clear evidence of potential negative effects. Of course, every individual is unique and different, and each case needs to be assessed individually. While the choice of whether bilingualism/multilingualism is appropriate for a given individual can be made on a case by case basis, it is time to change the notion that non-TD individuals should be discouraged a priori from embracing additional languages.

## Data Availability

The datasets generated during and/or analysed during the current study are available from the corresponding author on reasonable request.

## Footnotes

1 Succinctly, full-scale IQ is a comprehensive cognitive score that includes both verbal and nonverbal/performance measures (Dawson, 2013). For a full review of how full-scale IQ scores are computed for the *Wechsler Intelligence Scales (WIS)* see Lange (2011).
